# High Interest in a Long-Acting Injectable Formulation of Pre-Exposure Prophylaxis for HIV in Young Men Who Have Sex with Men in NYC: A P18 Cohort Substudy

**DOI:** 10.1371/journal.pone.0114700

**Published:** 2014-12-11

**Authors:** Kathrine Meyers, Kristina Rodriguez, Robert W. Moeller, Ilana Gratch, Martin Markowitz, Perry N. Halkitis

**Affiliations:** 1 Aaron Diamond AIDS Research Center, New York, New York, United States of America; 2 Middlebury College, Middlebury, Vermont, United States of America; 3 Center for HIV, Identity, Behavior and Prevention Studies, New York University, New York, New York, United States of America; 4 Global Institute of Public Health and Department of Population Health, New York University, New York, New York, United States of America; Centers for Disease Control and Prevention, United States of America

## Abstract

**Objective:**

In the context of continued high rates of condomless anal intercourse and HIV-1 infection, young men who have sex with men (YMSM) need additional effective and desirable HIV prevention tools. This study reports on the willingness of a racially-ethnically diverse cohort of YMSM to use a new biomedical prevention approach, a long-acting injectable pre-exposure prophylaxis (LAI-PrEP) agent.

**Methods:**

A cross-sectional study conducted between June-August 2013 recruited participants from an ongoing cohort study of YMSM in NYC. Participants included 197 YMSM, of whom 72.6% (n = 143) identified as men of color. Two outcomes were measured through computer-assisted self-interviews: 1) willingness to use long-acting injectable PrEP and 2) preference for route of administration of PrEP. In addition, concerns about perceived impacts of PrEP on health and risk behavior, access to health services, and stigma were investigated.

**Results:**

Over 80% (n = 159/197, p<0.001) of participants stated they would be willing to use LAI-PrEP. With regards to preference for mode of delivery 79.2% (n = 156/197, p<0.001) stated they would prefer an injection administered every three months over a daily pill or neither one.

**Conclusions:**

This study is the first to explore acceptability of LAI-PrEP in the US. A significant majority of participants expressed willingness to use LAI and the majority preferred LAI-PrEP. LAI-PrEP holds great promise in that it could circumvent the adherence challenges associated with daily dosing, especially if nested within appropriate psycho-behavioral support. Medical providers whose patients include YMSM at high risk for HIV infection should note the positive attitudes toward PrEP, and specifically LAI-PrEP.

## Introduction

Since research into an effective HIV prevention pill began, dozens of studies have queried potential target users on their willingness to take a daily dose of anti-retroviral drugs to protect themselves from acquiring HIV. Studies have reported on willingness to use such a product, [1]–[9] factors associated with interest in a daily oral prevention pill, [10], [11] and target users' concerns with a daily drug as a prevention intervention [3], [12]–[15]. Studies reporting on the acceptability of daily oral pre-exposure prophylaxis (PrEP) among men who have sex with men (MSM) have documented a wide range of acceptability, from as little as 28% to as high as 96%. [3], [6] These studies sought to identify demographic and behavioral factors, notably sexual risk factors, associated with a higher interest in daily oral PrEP. Following the US Food and Drug Administration's July 2012 approval of the once-daily combination of tenofovir disoproxil fumarate and emtricitabine (Truvada) as PrEP, these studies have provided a useful body of knowledge to inform the design and implementation of PrEP demonstration projects.

Building on this literature, our study set out to extend these research questions to explore willingness to use a long-acting injectable formulation of PrEP. Given the disproportionate effect of the HIV epidemic on young men who have sex with men (YMSM) of color in the US and especially in NYC, [16]–[18] we set out to report on willingness to use long-acting injectable PrEP (LAI-PrEP) in an ethnically-diverse cohort of YMSM in NYC. Additionally, we explored demographic and behavioral characteristics that may be associated with willingness to use LAI-PrEP and sought to determine preferences for one form of administration (injectable) over another (daily pill). These questions are relevant as LAI-PrEP appears to be a promising new intervention and its acceptability has not previously been explored in any US populations. Given positive results in the drug development pathway of long-acting injectable products, behavioral research to investigate willingness to use LAI-PrEP among potential users is timely. Recent studies have shown that a long-acting integrase-inhibitor, GSK 744, demonstrated 100% prevention efficacy in macaques against repeat low-dose rectal challenges with an HIV-related virus to which monkeys are susceptible. Notably, this new agent yielded a pharmacokinetic profile that suggested once-quarterly dosing maintained suitable levels of the drug to protect against HIV in humans[19]. Drug safety and tolerability studies in humans are underway, with the potential for efficacy trials to be conducted in the coming years. If effective, LAI-PrEP may be able to circumvent some of the adherence issues associated with the daily oral regimen [12], [20], [21], such as remembering to take medication daily, pill fatigue over time, or unintended disclosure of PrEP use to partners [12], [21], [22].

Should LAI-PrEP prove effective, safe, and acceptable, it has the potential to greatly impact the HIV epidemic, particularly in individuals engaging in behaviors that may increase their risk of HIV acquisition and who are seeking an alternative to daily oral PrEP. The aim of this exploratory study is to investigate interest in and attitudes towards LAI-PrEP. We hypothesized that young HIV-uninfected MSM would be more interested in LAI-PrEP than in a daily oral PrEP regimen.

## Methods

### Sampling and Recruitment

For this study two hundred participants were recruited from the emerging adult cohort study, Project 18 (P18), between June and August 2013. P18 is a longitudinal study conducted by the Center for Health, Identity, Behavior and Prevention Studies at New York University [23]. The P18 cohort enrolled young men age 18 to 19 years between 2009-2011, who lived in New York City, reported having sex with at least one man in the previous six months, and self-reported negative HIV serostatus. We contacted HIV-negative members of the P18 cohort and provided information about the current study through email, phone calls and text messages until 200 were enrolled. The composition of this cohort was comparable to that of the P18 cohort from which participants were sampled. Each participant was compensated $30 for time and travel costs. For further description of the P18 cohort, see Halkitis 2012 [23].

### Procedures

A trained interviewer introduced the study aims and provided a brief description of both daily oral and LAI-PrEP. The interviewer provided information on possible side effects of oral and LAI-PrEP (fatigue, head ache, nausea, diarrhea, vomiting), potential long-term health risks associated with taking the drug (impaired kidney function, decreases in bone density), and efficacy estimates with optimal adherence. For LAI-PrEP only, the possibility of pain at injection sites was also mentioned. Informed consent was obtained from all participants. To ensure confidentiality, participants entered their data directly into a computer-based questionnaire. The study, including all measures and procedures, was approved by the NYU Institutional Review Board.

### Measures

#### Outcomes

To assess preference for mode of PrEP administration respondents were asked “If you had a choice to use a daily pill or a shot every three months to protect you from HIV, which would you choose?” Participants chose one of four answers: prefer oral, prefer shot, neither, or uncertain. Due to the small numbers in the oral, neither and uncertain categories, we combined them to create a dichotomous variable which compared them against those who preferred LAI-PrEP.

#### Independent variables

Demographic variables: Mean age of all participants was calculated. Race and ethnicity was categorized into 5 distinct groups: Hispanic/Latino, Black Non-Latino, Mixed Race, White Non-Latino and Asian Pacific Islander (API) and other, which were collapsed due to the small number of participants in each category. Studies have shown that for younger participants asking about familial socioeconomic status (SES) is a better representation of their actual SES.[24] Therefore due to the overall youth of the cohort, participants were asked: “What do you perceive your family's socio-economic class to be?” (lower, lower middle, middle, upper middle or upper). Additionally, participants were asked about sexual behavior (MSM or MSM and MSW) and sexual orientation (gay or bisexual). Nativity (U.S. born or not), insurance status (yes or no) and self-perceived health (excellent, good, fair, poor) were also queried. Level of education was collected as some high school, completed high school, some college/technical school, college graduate, post-graduate.

Sexual behavior, substance use and STI variables: In order to assess risk behavior, participants were asked to recall the number of partners in the last three months and condom use frequency (all the time, almost all the time, sometimes, hardly ever, never). Participants were also asked to report any condomless receptive anal intercourse in the last three months. Substances utilized during the most recent sexual encounter with a casual and/or main partner were also queried. To assess history of sexually transmitted infections (STI), participants were asked to review a comprehensive list of STIs and check off any with which they had ever been diagnosed.

Concerns and interest: To better understand the pattern of reasoning and decision making around interest in LAI-PrEP use we explored concerns as well as questions related to service-delivery that might impact a participant's interest in LAI-PrEP. As no published studies to date have looked at concerns around LAI-PrEP, we adapted questions that have been asked in oral PrEP studies [1], [8], [25]. Participants were asked to state their level of agreement on statements listing concerns about the impact of PrEP use on health, behavior, stigma, and interest in psycho-behavioral support services to PrEP. For these items we used a five-point Likert scale (strongly agree to strongly disagree).

### Data Analysis

Descriptive statistics for demographics, sexual behaviors, concerns and interests about PrEP were generated. Chi-square, and when appropriate a fisher's exact test, were used to test independent associations between outcomes and demographic and behavioral predictors.

We modeled each of the two outcomes with bivariate analysis using demographic and behavioral factors as well as concerns that have been previously reported to have an association with willingness to use oral PrEP. We then performed a multivariable analysis on willingness to use LAI-PrEP. Given the small sample size, the multivariable model included factors that were significant at p<0.10 level in the bivariate model. The bivariate analysis for preference for modality of LAI-PrEP is not shown due to lack of significant associations at the p<0.10 level. We considered concerns voiced by ≥70% of the cohort to be indicative of group-level concern.

## Results

### Demographics

Of 200 participants enrolled, three self-identified as heterosexual and reported no sexual relations with a man in the last 12 months and were excluded from analyses, limiting the sample to 197 YMSM.

The young men in this cohort had a mean age of 21.2 (SD = 0.8). Given the study's focus on YMSM, and the tight age distribution, age was not treated as an independent variable. The population was racially, ethnically and socioeconomically diverse. Overall, 72.6% (n = 143) were men of color. Perceived familial socioeconomic status (SES) was well distributed across all income categories with the highest proportion reporting lower income (40.1%, n = 79). The cohort was well-educated, with over 76% having attended or graduated from college or technical school. Sexual behavior and sexual identity were comparable, with almost an equal number of men reporting having sex with men only (86.3%, n = 170) and identifying as gay (85.8%, n = 169). Almost 90% (n = 175) reported being in good or excellent health. Out of the nearly 80% (n = 157) who were insured, over half (53.8% n = 106) were covered by their parents' insurance plan (Table 1).

**Table 1:** Basic Demographics.

	Total
	N (%)
**Overall**	**197 (100)**
**Demographics**	
**Race**	
Hispanic/Latino	79 (40.1)
Black Non-Latino	34 (17.3)
API/Other	12 (6.1)
Mixed Race	18 (9.1)
White	54 (27.4)
**Perceived familial SES**	
Lower	79 (40.1)
Middle	73 (37.1)
Upper	45 (22.8)
**Education**	
High school or less	46 (23.4)
Some college/technical school	97 (49.2)
College degree or more	54 (27.4)
**U.S. born**	
Yes	177 (89.9)
No	20 (10.2)
**Sexual orientation**	
Gay	169 (85.8)
Bisexual	28 (14.2)
**Insured**	
Yes	157 (79.7)
No	40 (20.3)
**Self-perception of health**	
Fair	22 (11.2)
Good	109 (55.3)
Excellent	66 (33.5)
**Sexual behavior**	
**Number of partners in the last 3 months**	
0 partners	28 (14.4)
1 partner	62 (31.8)
2 or more partners	105 (53.9)
**Any condomless receptive anal sex in the last 3 months**	
Yes	74 (37.6)
No	123 (62.4)
**Any drug use during last sexual encounter**	
Yes	84 (42.6)
No	113 (57.4)
**Previous STI diagnosis**	
Yes	52 (26.4)
No	145 (73.6)
**Condom use frequency**	
Hardly ever/Never	15 (7.6)
Sometimes	31 (15.7)
Almost all the time	108 (54.8)
All the time	43 (21.8)
**Fear of HIV**	
Not afraid	22 (11.2)
Indifferent	18 (9.1)
Afraid	157 (79.7)

### Sexual behavior

There was a wide distribution of the number of male sexual partners in the last three months (from 0 to 89) with more than half reporting more than two partners (53.9%, n = 105). Over 70% (n = 139) reported inconsistent condom use while 7.6% (n = 15) reported hardly ever or never using condoms. In the last three months, more than a third reported having had condomless receptive anal sex (37.9%, n = 69) and 42.6% (n = 84) reported using some variety of drug on the day of their last sexual encounter. Over a quarter (26.4%, n = 52) had been diagnosed with at least one STI (Table 1).

### Willingness to use LAI-PrEP and preferred mode of administration

**Figure 1:**
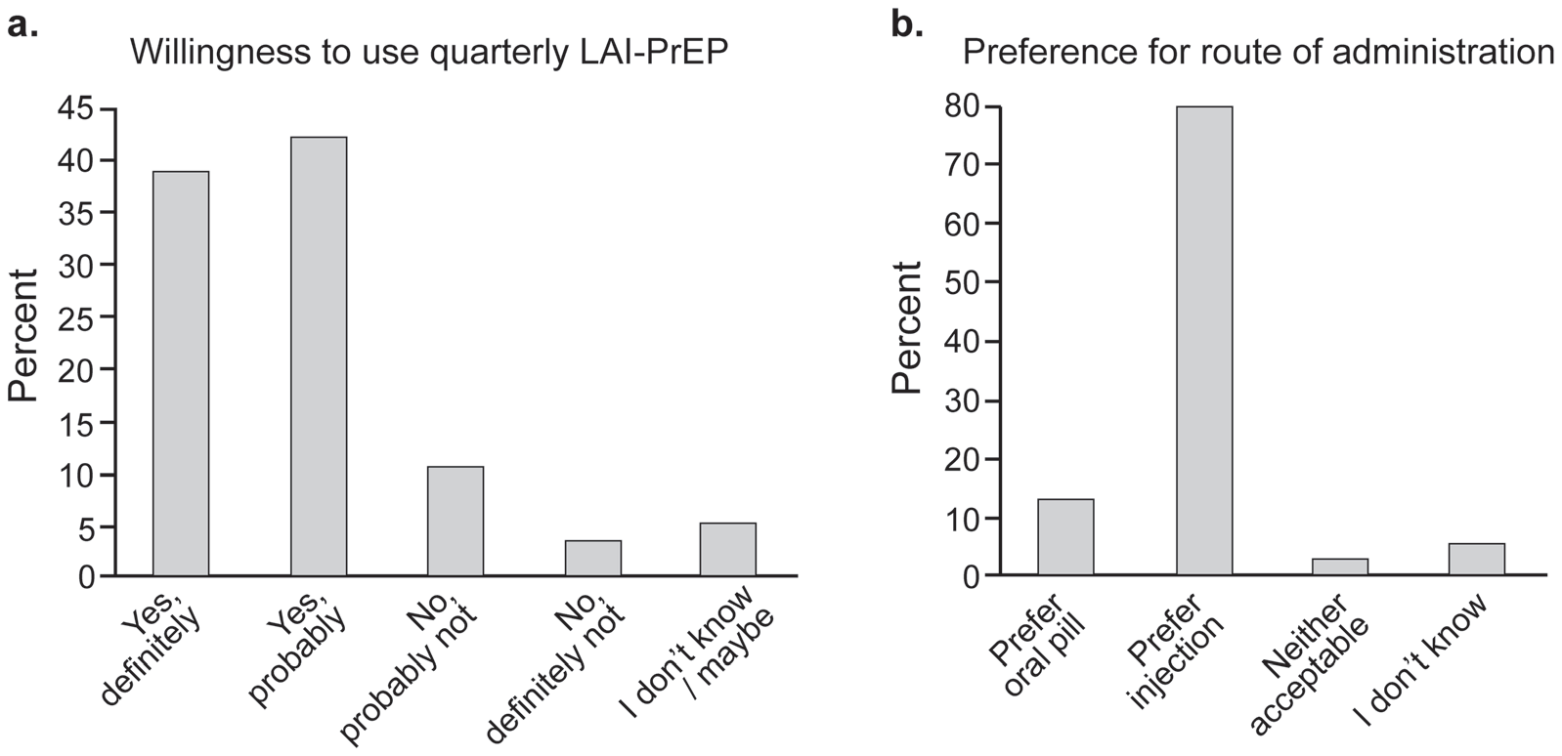
Distribution of main outcomes.

Over 80% (159/197) of participants stated that they would definitely or probably be willing to receive LAI-PrEP if it could effectively prevent HIV infection. (Fig. 1a and Table 2) Higher SES corresponded with lower willingness to use LAI-PrEP (χ^2^ = 5.38, df = 2, p = 0.07), as did some college education (χ^2^ = 10.78, df = 2, p = 0.004) (Table 2). Two measures of sexual risk behavior suggested higher willingness to use LAI-PrEP (history of an STI, χ^2^ = 2.73, df = 1, p = 0.10 and greater number of partners, χ^2^ = 4.82, df = 2, p = 0.09). Exploring further, the bivariate model suggested that compared to those with a college or post-graduate degree, those with some college or technical school had higher odds of being willing to try LAI-PrEP (OR = 3.9, 95% CI: 1.7-9.1), however there was no linear trend across level of education (Table 2). Those who cited having no partners in the last three months had lower odds of being willing to use LAI-PrEP as compared to those who reported two or more partners (OR = 0.4, 95% CI: 0.1, 0.9). Having previously been diagnosed with an STI showed borderline significance (OR = 2.2, 95% CI: 0.9 – 5.5). In the multivariable model education and number of partners retained significance at p<0.10.

**Table 2:** Willingness to use LAI-PrEP by demographics and sexual behavior (n = 197) and bivariate and multivariable analysis of willingness to use LAI-PrEP (n = 197).

	Willing to use	Not willing to use	p-value	OR (95%CI)			
	n (%)	n (%)		Bivariate		Multivariable	
Total	159 (80.7)	38 (19.3)	<0.001				
**Demographics**							
**Race**							
Hispanic/Latino	60 (37.7)	19 (50.0)	0.62	0.7	(0.3, 1.7)		
Black Non-Latino	29 (18.2)	5 (13.2)		1.3	(0.4, 4.3)		
API/Other	11 (6.9)	1 (2.6)		2.5	(0.3, 21.7)		
Mixed Race	15 (9.4)	3 (7.9)		1.1	(0.3, 4.7)		
White	44 (27.7)	10 (26.3)		-	-		
**Perceived familial SES**							
Lower	69 (43.4)	10 (26.3)	0.07	1.5	(0.5, 4.1)		
Middle	53 (33.3)	20 (52.6)		0.6	(0.2, 1.4)		
Upper	37 (23.3)	8 (21.1)		-	-		
**Education**							
High school or less	37 (23.3)	9 (23.7)	0.004	2.1	(0.8, 5.2)	2.1	(0.8, 5.7)
Some college/technical School	86 (54.1)	11 (29.0)		**3.9**	**(1.7, 9.1)**	**3.7**	**(1.5, 8.6)**
College degree or more	36 (22.6)	18 (47.4)		-	-	-	-
**U.S. born**							
Yes	140 (88.0)	37 (97.4)	0.13*	0.2	(0.02, 1.5)		
No	19 (12.0	1 (2.6)		-	-		
**Sexual orientation**							
Gay	139 (87.4)	30 (79.0)	0.20*	1.9	(0.7, 4.6)		
Bisexual	20 (12.6)	8 (21.0)		-	-		
**Insured**							
Yes	128 (80.5)	29 (76.3)	0.65*	1.3	(0.6, 3.0)		
No	31 (19.5)	9 (23.7)		-	-		
**Self-perception of health**							
Fair	20 (12.6)	2 (5.3)	0.43	2.7	(0.6, 12.9)		
Good	87 (54.7)	22 (57.9)		1.1	(0.5, 2.3)		
Excellent	52 (32.7)	14 (36.8)		-	-		
**Sexual behavior**							
**Number of partners in the last 3 months**							
0 partners	19 (12.0)	9 (24.3)	0.09	**0.4**	**(0.1, 0.9)**	**0.4**	**(0.2, 1.1)**
1 partner	49 (31.0)	13 (35.1)		0.6	(0.3, 1.4)	0.7	(0.3, 1,7)
More than 2 partners	90 (57.0)	15 (40.5)		-	-	-	-
**Any condomless receptive anal sex in the last 3 months**							
Yes	61 (38.4)	13 (34.2)	0.63	1.2	(0.6, 2.5)		
No	98 (61.6)	25 (65.8)		-	-		
**Any drug use during last sexual encounter**							
Yes	71 (44.7)	13 (34.2)	0.24	1.6	(0.7, 3.3)		
No	88 (55.4)	25 (65.8)		-	-		
**Previous STI diagnosis**							
Yes	46 (28.9)	6 (15.7)	0.10	**2.2**	**(0.9, 5.5)**	1.9	(0.7, 5.0)
No	113 (71.1)	32 (84.2)		-	-	-	-
**Condom use frequency**							
Hardly ever/Never	10 (6.3)	5 (13.2)	0.22	0.7	(0.2, 2.5)		
Sometimes	25 (15.7)	6 (15.8)		1.4	(0.5, 4.4)		
Almost all the time	92 (57.9)	16 (42.1)		2.0	(0.8, 4.7)		
All the time	32 (20.1)	11 (29.0)		-	-		
**Fear of HIV**							
Not afraid	19 (12.0)	3 (7.9)	0.47	1.7	(0.5, 6.0)		
Indifferent	16 (10.1)	2 (5.3)		2.1	(0.5, 9.7)		
Afraid	124 (78.0)	33 (86.8)		-	-		

* Fishers exact test was used.

**Bold** indicates significance

When participants were prompted to state their preference for mode of delivery of PrEP, a significant majority, 79.2% (n = 156, p<0.001), stated they would prefer an injection administered every three months (Fig. 1b). While preference for LAI-PrEP was significant no demographic or behavioral factors were found to be associated with a preference for LAI-PrEP over daily oral PrEP (data not shown).

### Concerns about PrEP

We grouped participants' concerns into four categories: health, behavioral, stigma, and psycho-behavioral support services (Table 3). Overall, the highest proportion of respondents (87.8%, n = 173) voiced worries over side effects, followed by long-term health effects (85.8%, n = 169), and reservations that PrEP would not be fully protective (83.3%, n = 164). Less than half of respondents (47.2%, n = 93) expressed concern that they might be more likely to have anal sex without a condom if taking PrEP. Reservations around stigma related to HIV infection and PrEP were lower than other issues, however still present with almost a third (28.4%, n = 56) of respondents concerned with being mistaken as HIV-infected and just over a third (35.0%, n = 69) fearing that others would want to know why they are taking PrEP.

**Table 3:** Concerns about PrEP (N = 197).

	Agree	Neither agree nor disagree	Disagree
	% (N)	% (N)	% (N)
**Health concerns**			
I am concerned about the side effects of PrEP.	173 (87.8)	17 (8.6)	7 (3.6)
I am concerned about the long-term effects of PrEP on my health.	169 (85.8)	18 (9.1)	10 (5.1)
I am concerned that if I do become HIV+, certain medicines won′t work because I was taking PrEP.	135 (68.5)	38 (19.3)	24 (12.2)
I am concerned that PrEP does not provide complete protection against HIV.	164 (83.3)	22 (11.2)	11 (5.6)
**Behavioral concerns**			
I am concerned about having to take a pill every day	101 (51.3)	47 (23.9)	49 (24.9)
I am concerned that taking PrEP means I am putting myself at risk for HIV.	81 (31.1)	42 (21.3)	74 (37.6)
I am concerned that taking PrEP might make me more likely to have anal sex without a condom.	93 (47.2)	37 (18.8)	67 (34.0)
**Stigma concerns**			
I am concerned that people will see me taking medication and think I have HIV.	56 (28.4)	41 (20.8)	100 (50.8)
I am concerned that people will see me taking medication and will want to know why I am taking it.	69 (35.0)	47 (23.9)	81 (41.1)
I am concerned about having to talk to my doctor about my sex life.	37 (18.8)	45 (22.8)	115 (58.4)
**Concerns about access and ancillary services**			
I would be more interested in PrEP if I did not have to pay for it.	157 (79.7)	29 (14.7)	11 (5.6)
I would be more interested in PrEP if I could get free HIV testing.	141 (71.6)	35 (17.8)	21 (10.7)
I would be more interested in PrEP if I could get access to free sexual health care/monitoring while taking PrEP.	157 (79.7)	23 (11.7)	17 (8.6)
I would be more interested in PrEP if I did **not** have to go to my regular doctor to get PrEP.	104 (52.8)	54 (27.4)	26 (13.2)
I would be more interested in PrEP if I could have access to one-on-one counseling and support around PrEP use.	146 (74.1)	32 (16.2)	19 (9.6)
I would be more interested in PrEP if I could get text based support for PrEP use.	117 (59.4)	52 (26.4)	28 (14.2)
I would be more interested in PrEP if I could talk to someone and get support or counseling about my sex life.	117 (59.4)	54 (27.4)	26 (13.2)
I would be more interested in PrEP if I could have group-based adherence support for PrEP use.	81 (41.1)	65 (33.0)	51 (25.9)

While insurance coverage in this cohort was high, more than half of the participants (52.8%) responded that they would be more interested in PrEP if they did not have to obtain the drug from their regular doctor. In addition, access to sexual health and psycho-behavioral support services, including free HIV testing, free sexual health care and counseling related to sexual health, PrEP, and HIV were attractive to respondents. On each of the measures above, over 70% agreed that they would be more interested in PrEP if it were accompanied by various psycho-behavioral support services (Table 3). The bivariate model demonstrated a number of associations between a participant's willingness to use LAI-PrEP and agreement with statements that proposed various psycho-behavioral support services to be delivered together with PrEP (Table 4). For instance, those who agreed that they would be more interested in PrEP if it were accompanied by free sexual health services, one-on-one counseling around PrEP use and sexual health, text-based support, or if it were free, all had significantly higher odds of being willing to use LAI-PrEP (with ORs ranging from 3.0 to 4.6) than those who disagreed with those statements (Table 4). However in the multivariable model no factors retained significance, despite the fact that we saw no evidence of multicollinearity in the multivariable regression.

**Table 4:** Bivariate analysis of interest in psycho-behavioral support services and willingness to use LAI-PrEP (N = 197).

	OR (95% CI)	
Characteristics		
I would be more interested in PrEP if I could get free HIV testing.		
Agree	2.4	(0.9, 6.7)
I don′t care	2.0	(0.6, 6.8)
I would be more interested in PrEP if I could get access to free sexual health care/monitoring while taking PrEP.		
Agree	**3.0**	**(1.0, 8.9)**
I don′t care	1.0	(0.3, 3.8)
I would be more interested in PrEP if I did not have to go to my regular doctor to get PrEP.		
Agree	1.2	(0.5, 3.0)
I don′t care	0.9	(0.3, 2.3)
I would be more interested in PrEP if I could have access to one-on-one counseling and support around PrEP use.		
Agree	**3.7**	**(1.3, 10.2)**
I don′t care	3.2	(0.9, 11.2)
I would be more interested in PrEP if I could get text based support for PrEP use.		
Agree	**4.1**	**(1.6,10.6)**
I don′t care	1.5	(0.6, 4.0)
I would be more interested in PrEP if I could talk to someone and get support or counseling about my sex life.		
Agree	**3.1**	**(1.2, 8.1)**
I don′t care	1.9	(0.7, 5.2)
I would be more interested in PrEP if I could have group-based adherence support for PrEP use.		
Agree	1.1	(0.5, 2.6)
I don′t care	1.4	(0.5, 3.4)
I would be more interested in PrEP if I did not have to pay for it.		
Agree	**4.6**	**(1.3, 16.3)**
I don′t care	1.9	(0.4, 7.7)

For each category the referent variable is Disagree.

## Discussion

A high percentage of the participants in this study expressed willingness to use a long-acting injectable form of PrEP. This preference appears to hold constant across demographic factors and sexual risk behaviors, though there is some suggestion that those who reported greater sexual risk, as captured by history of STI and number of partners, may be more willing to use LAI-PrEP. In addition, the analysis revealed that a high proportion of the participants preferred LAI-PrEP over the currently available regimen of a daily oral pill. We hypothesize that our inability to detect demographic and behavioral factors associated with preference for LAI-PrEP may be due to the unexpectedly small number of respondents who were not interested in LAI-PrEP (n = 41).

Blinded clinical trials, particularly iPREX, the only PrEP study conducted among MSM, have documented inconsistent results in PrEP efficacy mostly due to large differences in adherence to a daily pill. [26]-[28] As adherence to daily oral PrEP continues to be a challenge, the results from this study bode well for the continued development of LAI-PrEP. More specifically, the fact that LAI-PrEP only needs to be administered four times a year could mitigate challenges associated with daily adherence. While LAI-PrEP may mitigate some of these obstacles, a distinct set of issues, such as retention in care and persistence to injection visits over time, will likely arise and require further inquiry. In the future, studies will have to be conducted to understand these issues and develop appropriate models of service-delivery.

To date, only two published studies have asked target users about their interest in different PrEP modalities [1], [8] and to our knowledge this is the first study to explore attitudes and willingness to use LAI-PrEP in the US. Similar to our findings, one study querying Indian and Peruvian MSM, reported injections as the preferred route of PrEP administration, [1] while South African and Thai MSM preferred the daily oral pill [1], [8]. However, no further analyses on behavior or demographic characteristics associated with preference for LAI-PrEP were reported in these papers.

Of note, a high proportion of participants revealed some degree of apprehension related to the potential negative health effects of PrEP. It will be imperative that data on short and long term health effects is accurately captured from ongoing clinical trials and demonstration projects and disseminated to potential target users in ways that support informed decision-making around PrEP use.

Interestingly, participants expressed high interest in PrEP if the intervention were accompanied by sexual health services, support systems, and other health-related services, however the majority expressed preference to access PrEP from someone other than their primary care physician (Table 3). However, the concerns captured from the study suggest that even if PrEP is covered by insurance and available through general practitioners, PrEP acceptance may be enhanced when coupled with sexual health services and delivered as a biomedical intervention packaged together with psycho-behavioral support. For instance, adherence and persistence counseling will need to be patient-centered and may need to be offered in a range of modalities (one-on-one counseling, group-based counseling, text-based reminders) for different users. Health care workers will need to anticipate potential obstacles to adherence and persistence to PrEP, and develop strategies that can facilitate PrEP initiation and persistence for potential users [21]. Care providers will need to discuss sexual health in ways that address decisions around condom use/non-use, managing HIV risk as well as risk of other STIs, how to decide whether oral PrEP or LAI-PrEP is more suitable, and how to support decisions on starting and stopping PrEP. Lessons learned from the preferences in service delivery of first generation oral PrEP are likely to be relevant to the implementation of LAI-PrEP, if it proves to be effective.

Fear of decreased condom use has been a major undercurrent in discussions of daily oral PrEP. In this study, almost half of the participants voiced concerned that they might be more likely to engage in condomless sex if they were using PrEP. This differs from clinical trial settings, particularly in iPrEX, which showed that condom use increased over the trial period and that there was no evidence of risk disinhibition [26]. While it is possible that this may be attributable to participants' uncertainty whether they were receiving Truvada or a placebo due to randomization, the open–label extension study in which all participants received Truvada also failed to show a decrease in condom use [29]. Research is currently underway to study the question of the decrease in condom use within PrEP demonstration projects where all participants are receiving daily oral Truvada. The data from these studies should inform the development of realistic protocols to help health care professionals discuss decisions around condom use and non-use with potential daily oral PrEP users. Such findings will be equally relevant for potential LAI-PrEP users in the future.

Lastly, researchers have hypothesized that HIV-related stigma, which permeates the social context in which sex takes place, may impact the uptake of daily oral PrEP [12], [30] and our analysis uncovered that over a quarter of participants expressed concerns that people would presume that they have HIV. The fact that LAI-PrEP would be administered in the privacy of a clinic setting and would obviate the need for prescription bottles that could disclose PrEP use could be a significant advantage and could assuage these types of concerns. More research into stigma and venues for PrEP delivery is needed.

### Limitations

There are a number of limitations that should be recognized. The first was the high degree of interest in LAI- PrEP which limited variability and subsequently could account for the lack of statistical power needed to detect significant differences between behavioral and demographic factors associated with the outcomes. Second, the high degree of interest in LAI- PrEP found in this young and HIV-aware cohort may not be generalizable to other populations of MSM in the US or elsewhere. While the racial profile of this cohort matches closely the profile of those who are seroconverting in NYC, the participants in this study were relatively educated and may be more knowledgeable about HIV infection and prevention strategies than the general population. In addition, all participants were highly research-engaged subjects who access free HIV testing regularly and therefore may be more interested in the idea of PrEP than the target population. Despite the lack of generalizability to the general MSM population, capturing attitudes in this population is particularly important because YMSM of color are at highest risk for HIV infection and in need of additional and alternative prevention interventions. Another limitation, inherent in any instrument querying sexual behaviors, is a chance that social desirability bias would affect responses, particularly in two domains: self-report of risky behavior and interest in PrEP. All the young men in this cohort have been engaged in research through the P18 study for at least eighteen months and have a high degree of comfort and trust with the research team, as evident through their continued participation. Questions were answered privately on a computer so that we do not believe under-reporting of sexual risk would be likely. Social desirability response bias could have led to over-reporting of interest in PrEP, given the familiarity and existing relationship between participants and the research team. However we do not believe this bias would have impacted stated preference for injectable over daily oral administration, an important outcome of interest in the study.

Lastly, it is important to note that this study investigated YMSM's willingness to use LAI-PrEP. The authors acknowledge that intent does not predict actual future behavior. However, until such a product becomes available and actual uptake can be measured, hypothetical willingness to use LAI-PrEP is a useful measure to inform clinical development.

## Conclusion

PrEP through an injectable agent that could be administered four times a year holds great promise as an addition to the HIV prevention toolkit. Daily oral PrEP is building the foundation for its use as a novel HIV prevention approach by getting target users, the broader community, and health care providers acquainted with the idea of healthy people who are at elevated risk using ARVs to prevent acquisition of HIV. Daily PrEP demonstration projects will require the field to begin to address the challenges, like decisions around condom-use and adherence that will undoubtedly accompany the roll out of PrEP. It is our hope that for those individuals for whom adherence to a daily dosing regimen is not possible, a LAI-PrEP agent could be an effective alternative, especially if combined with appropriate psycho-behavioral support and appropriate ancillary services. The positive feedback from target users of LAI-PrEP captured in this study should encourage sociobehavioral researchers, public health workers, and general practitioners who serve at risk MSM to plan ahead to determine how best to deliver integrated biobehavioral PrEP to MSM at elevated risk for HIV acquisition [31]. Young MSM, particularly of color, are a subpopulation that account for a large proportion of infections in the US, and in NYC. If such young men are amenable to receiving an HIV prevention injection four times a year, the prospect for reducing the number of new infections among YMSM is promising.
